# Mesoscale Whole‐Brain *T*_2_ *‐Weighted and Associated Quantitative MRI in Humans at 10.5 T

**DOI:** 10.1002/mrm.70366

**Published:** 2026-04-07

**Authors:** Jiaen Liu, Peter van Gelderen, Jacco A. de Zwart, Jeff H. Duyn, Yujia Huang, Shuxian Qu, Andrea Grant, Edward J. Auerbach, Matt Waks, Russell L. Lagore, Lance Delabarre, Alireza Sadeghi‐Tarakameh, Yigitcan Eryaman, Gregor Adriany, Kamil Uğurbil, Xiaoping Wu

**Affiliations:** ^1^ Advanced Imaging Research Center UT Southwestern Medical Center Dallas Texas USA; ^2^ Radiology UT Southwestern Medical Center Dallas Texas USA; ^3^ Advanced MRI Section NINDS, NIH Bethesda Maryland USA; ^4^ Center for Magnetic Resonance Research, Radiology, Medical School University of Minnesota Twin Cities Minneapolis Minnesota USA

**Keywords:** 10.5 T, magnetic susceptibility *χ*, mesoscale whole‐brain MRI, *R*_2_* relaxation rate, *T*_2_*‐weighted MRI, ultrahigh field MRI

## Abstract

**Purpose:**

To demonstrate mesoscale whole‐brain *T*_2_*‐weighted (*T*_2_*w) MRI at 10.5 T, quantify *R*_2_* relaxation rate and magnetic susceptibility (*χ*), and evaluate *T*_2_*w contrast at such high field strength.

**Methods:**

Multi‐echo GRE (ME‐GRE) data were collected in healthy adults at 0.5 mm isotropic resolution at 10.5 T. Whole‐brain images were reconstructed with navigator‐guided joint motion and field correction and were used for quantitative *R*_2_* and *χ* mapping. Regional *R*_2_* and *χ* values and *R*_2_* contrast were analyzed in volumetric regions of interest (ROIs) and intra‐cortical surface‐based ROIs. For comparison, ME‐GRE data from the same subjects were acquired using a similar protocol at 7 T.

**Results:**

High‐quality whole‐brain *T*_2_*w images were obtained, enabling *R*_2_* and *χ* mapping with delineation of fine‐scale brain structures. Regional *R*_2_* analysis revealed a linear relationship between 10.5 T and 7 T *R*_2_* values with a slope of 1.52, in agreement with previously reported linear field dependency of *R*_2_*. Estimated *χ* values were field‐independent in most brain regions under consideration except for the basal ganglia where *χ* was observed to be lower at 10.5 T than at 7 T. The normalized *R*_2_* contrast that is, the *R*_2_* difference normalized by the mean *R*_2_*, increased by about 3% between brain regions and 12% between cortical depths from 7 to 10.5 T.

**Conclusion:**

It is feasible to achieve high‐quality mesoscale whole‐brain *T*_2_*w MRI at 10.5 T and associated quantitative *R*_2_* and *χ* mapping. Our results may aid future optimization of anatomic *T*_2_*w brain MRI at ultrahigh field beyond 7 T.

## 1 Introduction

The pursuit of mesoscopic resolution (i.e., ∼0.5 mm isotropic and better) in human brain imaging [1] in attempts to bridge the gap between macroscopic anatomical visualization and microscopic cellular details is a frontier in magnetic resonance imaging (MRI). Ultra‐high‐field (UHF) MRI systems operating at 7 tesla (T) and above unlock unprecedented signal‐to‐noise and contrast‐to‐noise ratios (SNR and CNR) [2, 3, 4, 5, 6, 7], enabling finer spatial resolution and enhanced sensitivity to mesoscopic tissue structures. Leveraging differences in tissue magnetic susceptibility (*χ*) to reveal intricate brain structures such as cortical layers [3, 8, 9, 10], brain vasculature [8, 11, 12, 13] and iron‐rich subcortical nuclei [3, 14], susceptibility‐weighted imaging, also known as *T*_2_*‐weighted (*T*_2_*w) imaging, particularly benefits from UHF MRI due to the synergistically enhanced SNR and susceptibility‐induced contrast with increasing field strength. When acquired with multi‐echo gradient‐echo (ME‐GRE) sequences, this modality along with its derived quantitative measurements, such as the transverse relaxation rate (*R*_2_* = 1/*T*_2_*) and *χ*, offers a window for studying brain anatomy [3] and pathology [15, 16, 17] in vivo at a spatial resolution previously attainable only ex vivo or with impractically long scan times [18]. The *T*_2_*w signal acquisition can be further accelerated using fast k‐space trajectories, such as segmented multi‐shot 3D echo‐planar imaging (EPI) [19, 20, 21] and spiral readouts [22, 23], in synergy with parallel imaging techniques [24, 25].

Human brain MRI at 10.5 T was demonstrated feasible [26], safe [27] and capable of delivering high SNR and parallel imaging performances with high‐density radiofrequency (RF) receive arrays [28, 29]. It holds promises to further improve achievable in vivo MRI resolution based on the *T*_2_*w signal beyond what has been achieved at 7 T [10, 30, 31]. However, there is a lack of literature reporting quantitative *R*_2_* and *χ* at 10.5 T and beyond [7, 26], obscuring the optimization of sequence parameters for realizing the full benefits of 10.5 T for *T*_2_*w MRI.

In this work, we reported *R*_2_* and *χ* based on ME‐GRE data with 0.5 mm isotropic resolution and whole brain coverage at 10.5 and 7 T in the same human subjects. At 10.5 T, we successfully implemented a motion‐robust ME‐GRE sequence, which was originally developed for 7 T applications [10, 17, 32], and a custom‐built high‐density 80‐channel receive RF coil [29] in our data acquisition. *R*_2_* and its inter‐regional contrast as well as *χ* differences between 10.5 and 7 T may shed light on exploiting the *T*_2_*w contrast for mesoscale MRI at field strengths at 10.5 T and beyond.

## 2 Methods

### 2.1 MRI Experiments

Four healthy human volunteers (three males and one female, 23–55 years old) were recruited and scanned in the MRI experiments after signing informed consent approved by the local Institutional Review Board. Data were first acquired on a Siemens MAGNETOM Dotplus 10.5 T MR scanner (Siemens Healthineers, Erlangen, Germany) equipped with 16‐channel RF transmission and 128‐channel signal reception systems. A custom‐built high‐density RF coil with 80 receive channels [29] was utilized for data collection. The 16 transmit channels of this coil were operated using predefined RF shimming to produce a transmit *B*_1_ pattern similar to the circularly polarized (CP) mode at 7 T. The same human volunteers were scanned at 7 T on a Siemens Terra MR scanner (Siemens Healthineers, Erlangen, Germany), using the commercial Nova single‐channel transmit 32‐channel receive RF coil (Nova Medical, Wilmington, MA, USA).

At 10.5 T, a 3D ME‐GRE sequence with embedded volumetric navigators was used for *T*_2_*w data acquisition [32]. Relevant imaging parameters included 0.5 mm isotropic resolution, 240 × 180 × 128 mm^3^ field of view (FOV), 35 ms repetition time (TR), 4 echo times (TEs), TE_1_ of 10.2 ms, echo spacing (ES) of 4.9 ms, 12° nominal flip angle (i.e., the Ernst angle assuming *T*_1_ of 1.75 s for the mean value across gray and white matter at 10.5 T [33]) and 208 Hz/pixel readout receiver bandwidth. Parallel imaging with controlled aliasing (CAIPI) [34] was applied with an acceleration factor of two in the phase‐encoding (left–right) direction and three in the slice‐encoding (head‐foot) direction, leading to a total scan time of ∼13.3 min.

To measure real‐time rigid‐body head motion and spatial *B*_0_ changes, low‐resolution volumetric navigator images were acquired in parallel with the high‐resolution GRE data. The navigator images were acquired using a segmented multi‐shot 3D EPI trajectory during the time period between RF excitation and GRE acquisition. The navigator EPI trajectory was accelerated by a factor of 4 × 2 (phase‐encoding × slice‐encoding) with blipped CAIPI [35]. More details about the navigator acquisition can be found in our previous work [10, 32]. In this work, the navigator images were acquired with 5 × 5.6 × 8 mm^3^ spatial resolution, 48 × 32 × 24 matrix size and 4.3 ms TE. Each navigator image volume was acquired in 12 TRs (420 ms).

At 7 T, *T*_2_*w data acquisition was performed using the same 3D ME‐GRE sequence as the 10.5 T experiments. Imaging parameters including FOV, resolution, 2D acceleration, TE_1_ and ES were matched to the 10.5 T protocol. Due to the longer *T*_2_* values, the 7 T data acquisition employed a 6‐echo protocol, leading to a longer TR of 45 ms, a higher nominal flip angle of 14° (using mean 7 T *T*_1_ of 1.5 s [33]), and a longer total scan time of ∼17.2 min. The volumetric navigator images were collected using the same imaging parameters as those of the 10.5 T protocol, except that their temporal resolution increased to 540 ms due to the increased TR.

For image reconstruction, the sensitivity maps of individual receive channels were obtained by acquiring fully sampled calibration data in a separate reference scan using a multi‐slice 2D GRE sequence. Specifically, multi‐slice 2D GRE images with whole brain coverage were obtained with the following parameters: 4 mm isotropic in‐plane resolution, 256 × 192 mm^2^ FOV, 4 mm slice thickness and 50 axial slices. At each field strength, TE was set to the minimum water‐fat in‐phase TE, 2.56 ms for 10.5 T and 2.88 ms for 7 T, for improved sensitivity map estimation. TR was set to 315 ms for 10.5 T and 500 ms for 7 T, and nominal flip angle to 30° for 10.5 T and 38° for 7 T, leading to a total scan time of less than 25 s.

For image processing and analysis, whole‐brain anatomical reference images were collected using *T*_1_‐weighted (*T*_1_w) 3D magnetization prepared two rapid acquisition gradient echoes (MP2RAGE) [36] at 7 T. MP2RAGE parameters included 0.7 mm isotropic resolution, 2.36 ms TE, 4 s TR, and TI_1_/TI_2_ = 740/2430 ms.

### 2.2 Image Reconstruction

All 3D *T*_2_*w ME‐GRE images were reconstructed with in‐house MATLAB (Mathworks, Natick, MA, USA) software (https://github.com/jiaen‐liu/moco). The reconstruction [10, 32] was based on a unified signal model incorporating intra‐scan rigid‐body motion and *B*_0_ changes for motion and *B*_0_ correction, and coil sensitivity maps for SENSE‐based parallel imaging reconstruction [37]. Coil sensitivity maps were estimated from the 2D GRE reference scans using an in‐house algorithm, including normalization of individual channel images by the channel‐combined image followed by spatial smoothing [32]. Motion time series was estimated from the navigator magnitude images using an iterative multi‐resolution image registration approach [38], whereas *B*_0_ changes over time were estimated based on the navigator phase images. The *T*_2_*w GRE images were reconstructed with joint motion and spatially linear *B*_0_ change correction [10, 32].

### 2.3 *R*_2_ * and *χ* Quantification

Before *R*_2_* calculation, the GRE magnitude data for each TE were corrected for the macroscopic *B*_0_ inhomogeneity. The corrected GRE magnitude signal for each TE was $\left|\hat{s}\left(\mathrm{TE}\right)\right|=\left|\frac{s\left(\mathrm{TE}\right)}{\sin c\left(\frac{\gamma \left|G\cdot ∆r\right|\mathrm{TE}}{2}\right)}\right|$, where ∆r defines the voxel size along the three axes [39] and **G** is the spatial gradient of B_0_. Voxel‐wise *R*_2_* values were calculated based on nonlinear least square fitting of the corrected complex ME‐GRE data, including unknowns of *R*_2_*, off‐resonant frequency and the signal phase at TE = 0. Complex fitting can avoid a negative bias in *R*_2_* estimation using only magnitude data when the signal SNR is low at long TEs [40].

For quantitative susceptibility mapping (QSM), voxel‐wise *χ* values were quantified using the pipeline implemented in the JHU/KKI QSM toolbox (https://github.com/xuli99/JHUKKI_QSM_Toolbox) [41, 42, 43]. Briefly, the QSM reconstruction included the following processes: path‐based phase unwrapping [44], brain masking using FSL BET [45], echo combination using weighted echo averaging [46], background field removal combining LBV [47] and VSHARP [48], and dipole inversion using a modified structural feature collaborative reconstruction approach [41] based on a nonlinear data fidelity cost function [49].

### 2.4 Image Processing and Data Analysis

For each subject, *T*_2_*w ME‐GRE magnitude, *R*_2_*, *χ*, and MP2RAGE images, were registered to the subject's 7 T *T*_2_*w GRE magnitude image space. This was done by aligning the 10.5 T *T*_2_*w magnitude and 7 T MP2RAGE with the 7 T *T*_2_*w magnitude image using multi‐contrast registration algorithms implemented in the Advanced Normalization Tools (ANTs) [50]. B‐spline interpolation was applied to minimize resolution loss during image transformation [51]. For alignment of *R*_2_* and *χ*, the same transformation matrix from the *T*_2_*w magnitude coregistration was applied.

Analysis of *R*_2_* and *χ* was performed based on volumetric regions of interest (ROI) and surface‐based ROIs at different cortical depths. Segmentation was performed on the skull‐removed MP2RAGE data in FastSurfer [52, 53]. Skull removal was performed on the second‐inversion image from the MP2RAGE sequence using the software BET [45]. FastSurfer generated FreeSurfer‐compatible ROIs including volumetric ROIs defined in “wmparc.mgz” and surface meshes residing on the pial surface and the gray and white matter boundary. Surface meshes at intermediate cortical depths were created using “mris_expand” in FreeSurfer [54].

In volumetric ROI‐based analysis, normalized *R*_2_* contrast, $\frac{{∆R}_{2}^{*}}{\bar{R_{2}^{*}}}$, was calculated for each pair of ROIs. For a given pair, ${∆R}_{2}^{*}$ was the difference between the two region‐average *R*_2_* values and $\bar{R_{2}^{*}}$ the mean *R*_2_* value across the two ROIs. In surface ROI‐based analysis, *R*_2_* contrast was evaluated between two surfaces at the 30% versus 80% cortical depths. Specifically, ${∆R}_{2}^{*}$ and $\bar{R_{2}^{*}}$ values were first calculated for each pair of corresponding vertices from the two surfaces. This preserves the depth‐dependent *R*_2_* variation in the contrast calculation. The median value of $\frac{{∆R}_{2}^{*}}{\bar{R_{2}^{*}}}$ in a given surface ROI was then calculated to evaluate the contrast in that ROI. Linear regression was performed by considering the variance in both variables using in‐house software developed based on Press et al. [55].

## 3 Results

**FIGURE 1:**
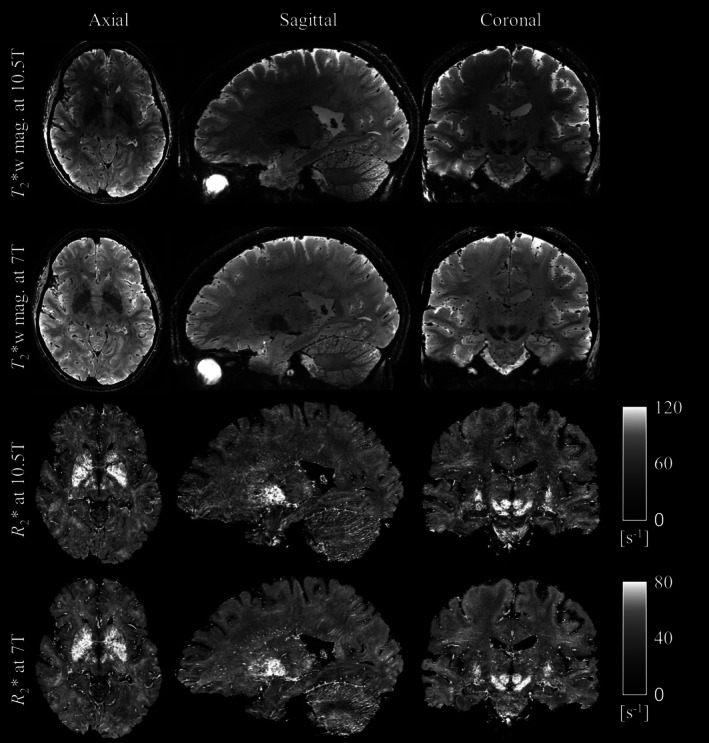
Example images of whole‐brain *T*_2_*‐weighted GRE magnitude and decay rate *R*_2_* maps in three orthogonal views from one subject scanned at 10.5 versus 7 T. The GRE magnitude images were the root mean square of individual echo magnitude images. Data were acquired with 0.5 mm isotropic resolution and reconstructed with navigator‐guided joint motion and *B*_0_ correction.

Examples of the echo‐averaged *T*_2_*w magnitude images and *R*_2_* maps from one subject are shown in Figure 1. Results from both field strengths exhibit high quality and detailed anatomical structures in the entire brain, including the cerebrum, brain stem and cerebellum. In the *T*_2_*w magnitude images, the 10.5 T data show increased intensity variation as expected from the increased transmit *B*_1_ inhomogeneity at this field strength. Overall, both *R*_2_* maps demonstrate consistent visual appearance across field strengths using gray scales adapted to each field.

**FIGURE 2:**
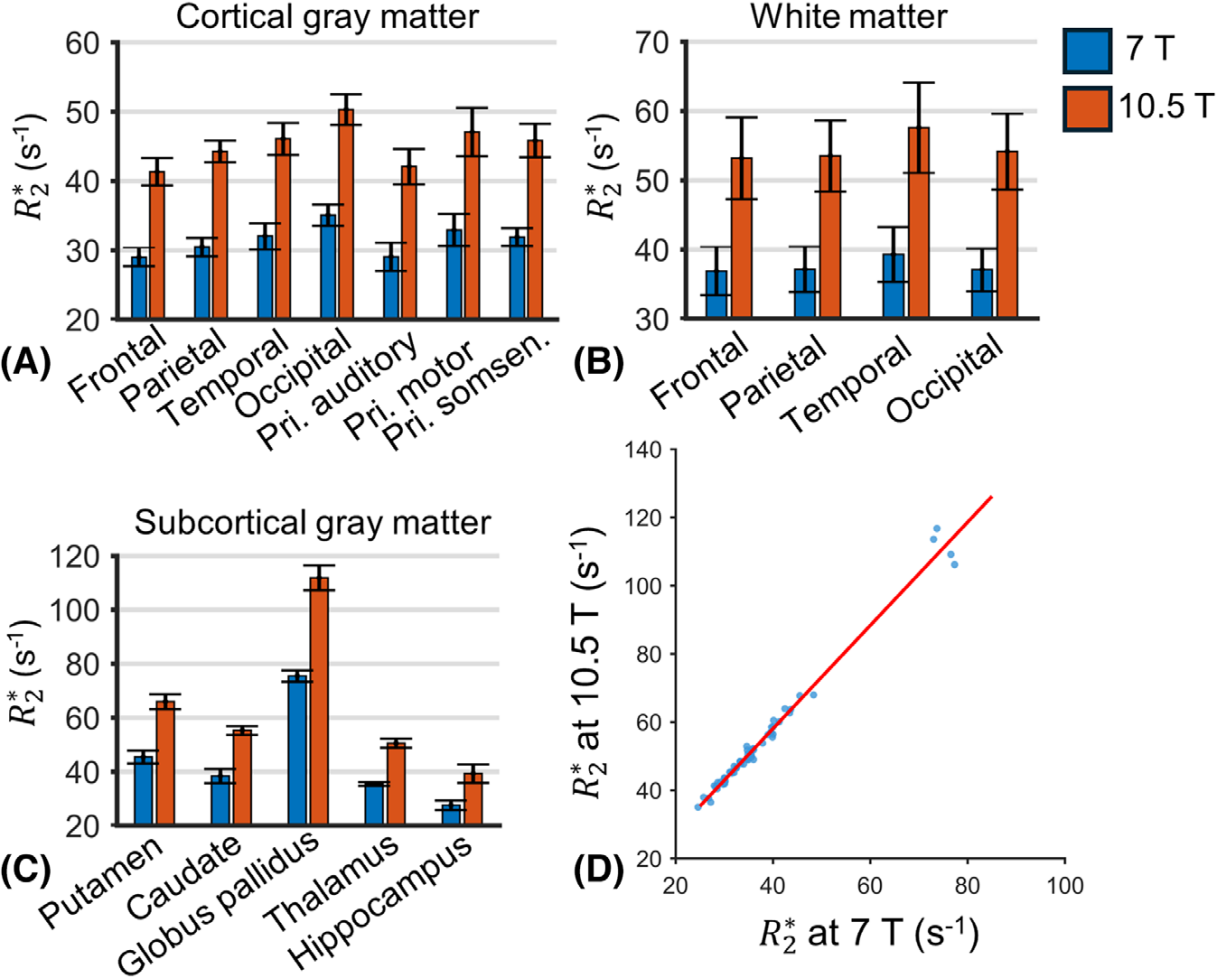
Group results of region‐averaged *R*_2_* in cortical gray matter (A), white matter (B) and subcortical gray matter regions (C), at 10.5 versus 7 T. In (A–C), shown are the group average *R*_2_* for each region and its standard deviation across subjects (*n* = 4). In (A), data from functional regions including the primary auditory cortex, primary motor cortex and primary somatosensory cortex are shown separately from the lobular cortical data. Relationship of the regional *R*_2_* values between the 10.5 and 7 T data is shown in (D), with each data point representing the region‐averaged *R*_2_* values from a single subject. The red solid line indicates the linear fit of the data.

In Figure 2, region‐averaged *R*_2_* data from the four subjects are summarized, including cortical gray matter in brain lobes and functional regions in Figure 2A, white matter in brain lobes in Figure 2B and subcortical gray matter regions in Figure 2C. Comparing Figure 2A with Figure 2B, it can be seen that the cortical gray matter regions demonstrate a higher level of *R*_2_* variation compared to the white matter regions. Overall, association cortex, such as the frontal lobe excluding the primary motor cortex and the parietal lobe excluding the primary somatosensory cortex, exhibits lower *R*_2_* values than cortical regions of primary sensory and motor functions, such as the occipital lobe and primary functional regions, in agreement with previous work [39, 56]. In Figure 2C, regions in the basal ganglia show the highest *R*_2_* value due to high iron concentration [57], compared to other subcortical regions including the thalamus and hippocampus.

The *R*_2_* variation demonstrates similar trends between 7 and 10.5 T. This observation can be quantitatively characterized by a linear relationship between the two as shown in Figure 2D. A linear regression of 10.5 versus 7 T *R*_2_* values revealed a slope of 1.52 ± 0.02 and intercept of −2.6 ± 0.9 s^−1^. Note that the slope closely matched the field strength ratio of 1.5, which can be a result of the linearly increasing *R*_2_* with field strength as previously reported [58, 59] (see discussions in Section 4 for more details).

**FIGURE 3:**
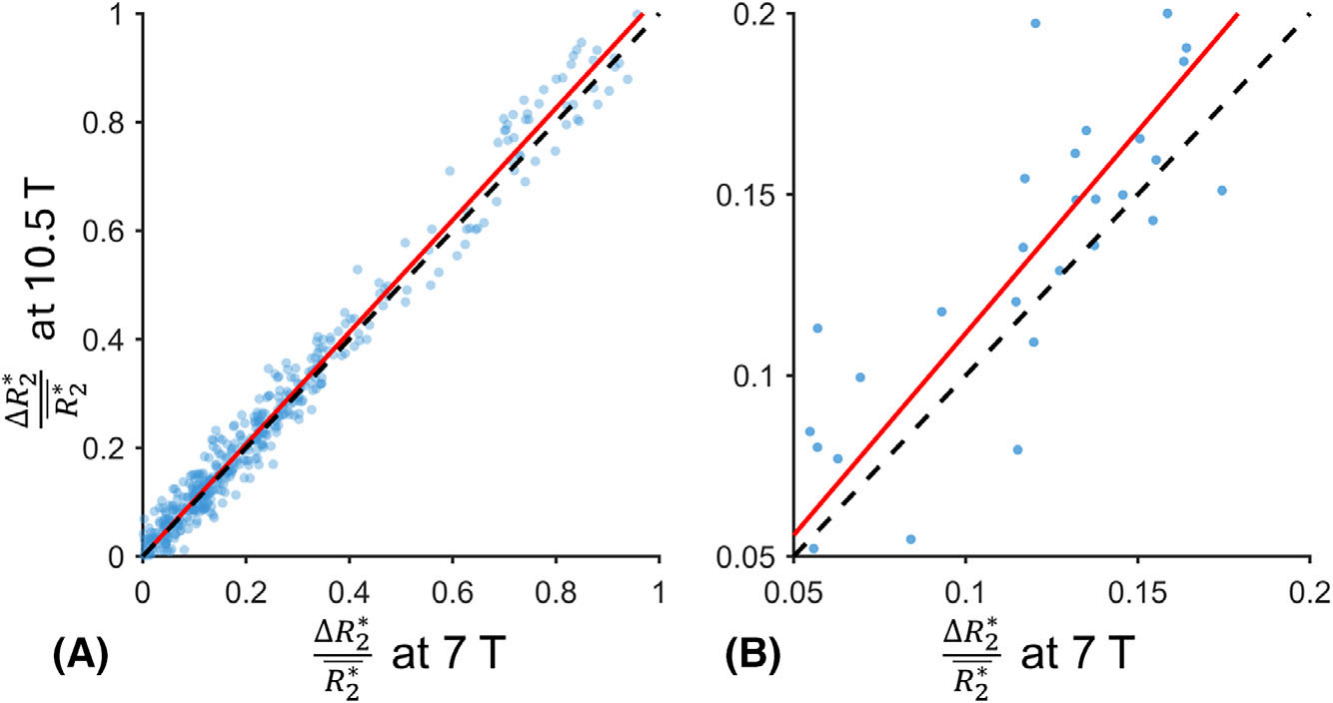
Relation of inter‐regional (A) and intra‐cortical (B) normalized *R*_2_* contrast ($\frac{{∆R}_{2}^{*}}{\bar{R_{2}^{*}}}$) between 10.5 and 7 T in all subjects (*n* = 4). In (A), each data point represents the normalized *R*_2_* contrast between a pair of regions shown in Figure 2 in a single subject. In (B), each data point represents the region‐averaged normalized *R*_2_* contrast between the 30% and 80% cortical depth from a single subject, where the included cortical regions are shown in Figure 2A. The red solid lines indicate the linear fit of the data, and black coarse dash lines mark the identity lines.

The normalized *R*_2_* contrast $\frac{{∆R}_{2}^{*}}{\bar{R_{2}^{*}}}$ between brain regions and cortical depths (30% vs. 80% depth) are summarized in Figure 3. A moderate increase of $\frac{{∆R}_{2}^{*}}{\bar{R_{2}^{*}}}$ at 10.5 T can be appreciated with the linear fit of 10.5 T versus 7 T data being slightly above the identity lines. Linear regressions without an intercept yielded a slope of 1.034 ± 0.005 for inter‐regional normalized *R*_2_* contrast (Figure 3A), and a slope of 1.12 ± 0.04 for the intra‐cortical normalized *R*_2_* contrast (Figure 3B). The results suggest that on average, the inter‐regional normalized *R*_2_* contrast increased by 3.4% ± 0.5%, and the intra‐cortical contrast by 12% ± 4%, from 7 T to 10.5 T.

**FIGURE 4:**
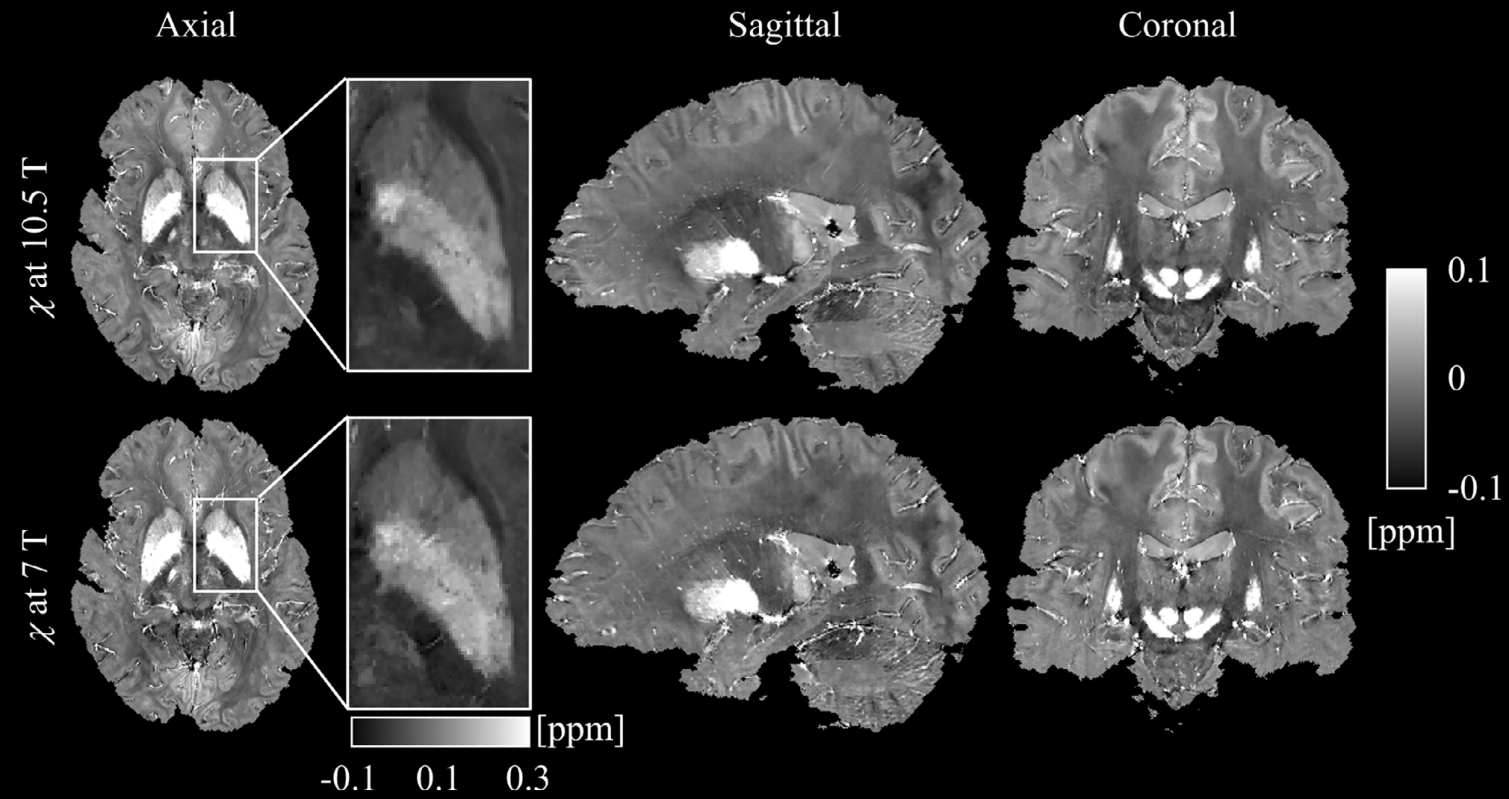
Example images of whole‐brain quantitative susceptibility (*χ*) mapping (QSM) in three orthogonal views from one subject scanned at 10.5 versus 7 T. Note that the *χ* maps surrounding the globus pallidus and putamen are enlarged using a different intensity scale for a more detailed view.

**FIGURE 5:**
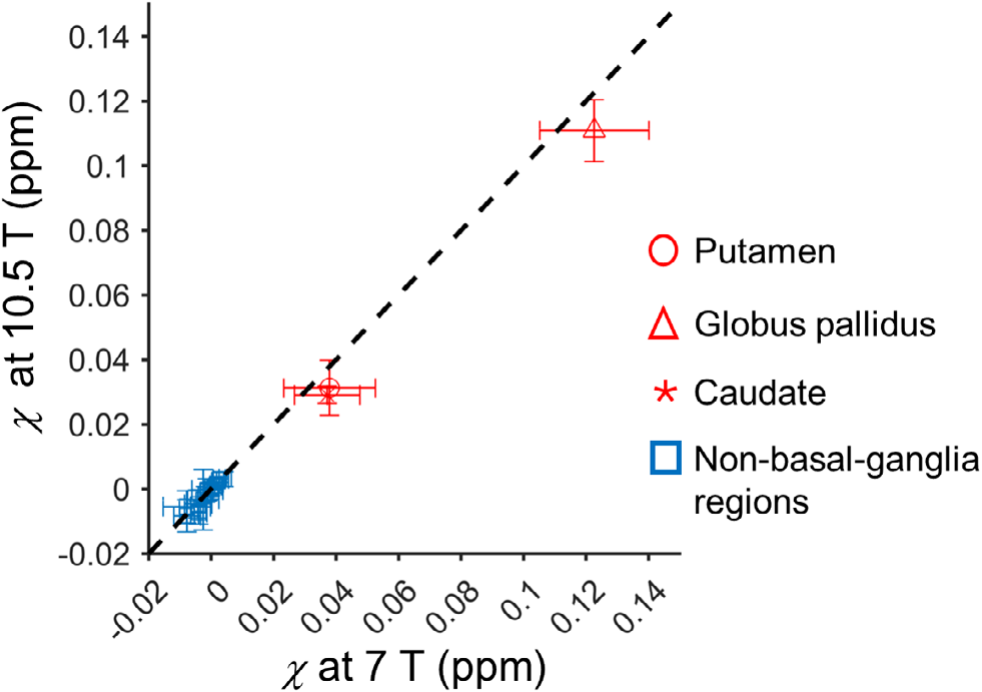
Relation of measured region‐averaged susceptibility (*χ*) between 10.5 and 7 T in all subjects (*n* = 4). Each data point represents the mean region‐averaged *χ* value across subjects with error bars indicating the standard deviation. The black dash line marks the identity line.

Figure 4 shows the reconstructed *χ* maps from the same subject included in Figure 1, demonstrating overall consistent values between 10.5 and 7 T as expected. Regional *χ* data at both field strengths from all subjects can be found in Figure 5. In the basal ganglia regions, such as the putamen, caudate, and globus pallidus, where the *χ* values are much higher compared to other regions, the measured *χ* values were slightly reduced at 10.5 T. In other regions, the values were consistent between the two field strengths.

## 4 Discussion

In this work, we acquired quantitative maps of *R*_2_* and susceptibility *χ* with 0.5 mm isotropic resolution and whole brain coverage in a group of human subjects (*n* = 4) at both 10.5 and 7 T. Our results provide important data for optimal design of *T*_2_*w MRI methods at such high field strengths.

At field strengths from 1.5 to 7 T, previous works [58, 59] have revealed that the *R*_2_* increase follows a nearly linear relationship with the field strength in different brain regions. A similar trend has also been observed in the *R*_2_ decay rate [60, 61]. These studies further demonstrated that the coefficients of the linear field dependency (slopes and intercepts) are region‐specific and related to the underlying tissue properties such as iron, lipid, and protein concentrations. In our data shown in Figure 2D, a strong linear relationship was observed between the regional *R*_2_* data at 10.5 and 7 T with a slope of 1.52, which is nearly identical to the field strength ratio, independent of tissue types. This can be a result of the continuation of the linear *R*_2_* trend observed at lower field strengths, which will lead to negligible contribution of the intercept to *R*_2_* value at very high field strength.

Another interesting finding was observed in the lower *χ* measured using QSM at 10.5 versus 7 T in subcortical regions (Figure 5). The apparently reduced *χ* compared to the 7 T results can be caused by the compartmental effects of water signal in adjacent white matter. As previously shown [62], water residing in axons, myelin sheath, and interstitial space demonstrates different signal decay rates and frequencies, which also depend on the fiber orientation. At higher field strengths, phase data in the white matter will be more biased towards water compartments with lower signal decay rates, and such bias will affect the reconstructed QSM in the subcortical gray matters. For example, near the globus pallidus, the phase data in the internal capsule can be shifted to the more positive direction at higher field strengths, due to the faster decay and negative frequency shift in the myelin water compartment when the fibers are in parallel with the filed [62]. Such effect may impact the QSM reconstruction of globus pallidus. This compartmental effect should also predict reduced *χ* reconstructed with longer echo times. Indeed, in our 7 T data, *χ* results from longer echo times (20–34.7 ms) in the same subcortical regions exhibit reduced value in comparison with the results reconstructed with shorter echo times (10.2–24.9 ms) (Figure S1). Similar echo‐dependent *χ* results have been observed previously in various brain regions [63]. It is possible that compartmental effect within the subcortical nuclei can contribute to field‐ and echo time‐dependent QSM. However, it requires more detailed simulation and experiment studies to confirm because of the more complicated microstructures in the gray matter than the white matter.

Magnetic saturation of biological forms of iron, such as ferritin, at 10.5 T has not been reported and can potentially contribute to the observed reduced *χ* at 10.5 T in the iron‐rich subcortical regions. Based on magnetization measurement of ferritin samples, no significant magnetic saturation has been observed at body temperature up to 5–7 T [64, 65]. If magnetic saturation were a significant driver, we should also expect the *R*_2_* increase from 7 to 10.5 T to be lower in iron‐rich regions than data shown in Figure 2D.

The optimal *T*_2_*w CNR per unit time, also known as the CNR efficiency, is achieved by using a TE equal to the *T*_2_* of the tissue of interest and TR scaled with TE. Based on the steady state formula of spoiled GRE signal assuming Ernst angles at TR < <*T*_1_, the CNR efficiency of *T*_2_*w signal is proportional to $\text{iSNR}\sqrt{\frac{R_{1}}{R_{2}^{*}}}\frac{∆R_{2}^{*}}{R_{2}^{*}}$ [2], where iSNR is the intrinsic SNR of the longitudinal magnetization at equilibrium and *R*_1_ is the longitudinal relaxation rate. This formula reflects that the overall CNR is determined by signal attenuation caused by reduced R_1_, constraint on noise averaging imposed by 1/*R*_2_* and the normalized *R*_2_* contrast $\frac{∆R_{2}^{*}}{R_{2}^{*}}$. Based on the linear trend of *R*_2_* following field increase, it has been predicted that $\frac{∆R_{2}^{*}}{R_{2}^{*}}$ will level off at sufficiently high field strength [2]. In our data shown in Figure 3, the inter‐regional and intra‐cortical $\frac{∆R_{2}^{*}}{{\bar{R}}_{2}^{*}}$ increased by about 3% and 12%, respectively, from 7 to 10.5 T, in agreement with the previous prediction. Therefore, our results suggest the crucial role of intrinsic SNR gain at 10.5 T for enhancing *T*_2_*w CNR. Recently, we have shown the increased intrinsic SNR particularly at the center of the coils based on phantom measurements at 10.5 T including the 80‐channel RF coil [29] used in this study and the 128‐channel RF coil in our previous work [28]. Importantly, the reduced parallel imaging penalty on SNR with high acceleration factors using these high‐density coils at 10.5 T is another advantage of increasing the field strength for MRI with very high resolutions [28]. It is worth noting that the current coil used at 10.5 T is the first generation of its kind. Our ongoing work demonstrates the potential of improved SNR in human experiments at 10.5 T. Future work will include a more comprehensive evaluation of the SNR and CNR gains of *T*_2_*w imaging at 10.5 versus 7 T.

One limitation of the current study was that all data collection at 10.5 T was performed by driving the RF coil in the CP‐like mode. The associated transmit *B*_1_ (*B*_1_^+^) inhomogeneity across the brain was more severe than that of the 7 T data, reducing SNR of *R*_2_* fitting especially in lower *B*_1_^+^ regions. But the *B*_1_^+^ inhomogeneity is not expected to introduce bias in the *R*_2_* fitting and the interpretation of the current results. Part of our future work is to address *B*_1_^+^ inhomogeneity by designing parallel transmission pulses with spatial spectral criteria [66] to achieve uniform water‐selective excitation across the brain without additional fat saturation pulses. Such pulses will also promote fast imaging with EPI and spiral k‐space trajectories, which are sensitive to artifacts caused by fat signals in high‐resolution *T*_2_*w protocols [13, 20, 23].

## 5 Conclusion

We demonstrated the feasibility of achieving high‐quality mesoscale whole‐brain *T*_2_*w GRE MRI at 10.5 T by combing a motion‐robust imaging technique with a high‐density RF coil. We also investigated potential gains in magnetic susceptibility contrast across a variety of brain regions by comparing against 7 T data obtained in the same subjects. At 10.5 T, *R*_2_* increases continued to follow the linear trend previously observed up to 7 T. Surprisingly, apparent *χ* values, expected to be independent of field strength, modestly decreased at 10.5 T in several subcortical regions. A potential explanation is the contribution from water compartments in tissue showing different decay rates and frequencies, weighted differently at specific field strengths and echo times. We also quantified the gain of the normalized *R*_2_* contrast $\frac{{∆R}_{2}^{*}}{R_{2}^{*}}$ between brain regions and cortical depths. Overall, our results provided important data for optimal design of *T*_2_*w MRI at 10.5 T and beyond.

## Funding

This work was supported by the National Institutes of Health (P41 EB027061, R01 NS136490, S10 RR029672, and U01 EB025144), National Institute of Neurological Disorders and Stroke (ZIA NS002990), Hamon Charitable Foundation, and Texas Instruments Foundation.

## Supporting information


**Figure S1:** Relation of measured region‐averaged 7 T susceptibility (*χ*) reconstructed using short vs. long echo times (TE) in all subjects (*n* = 4). Each data point represents the mean region‐averaged value across subjects with error bars indicating the standard deviation. The black dash line marks the identity line.

## Data Availability

The human brain data including ME‐GRE images at both 7 and 10.5 T alongside *T*_1_w MP2RAGE at 7 T are publicly available at https://openneuro.org/datasets/ds007418/. The MATLAB code for image reconstruction is made publicly available at https://github.com/jiaen‐liu/moco.
