# Supplementary material for: Mesoscale Whole‐Brain T 2 *‐Weighted and Associated Quantitative MRI in Humans at 10.5 T
Source: Magn Reson Med. 2026 Apr 7;96(2):817–25. doi: 10.1002/mrm.70366 (PMC13269191; doi:10.1002/mrm.70366)
Supplement: Supplementary file 1 — Figure S1: Relation of measured region‐averaged 7 T susceptibility (χ) reconstructed using short vs. long echo times (TE) in all subjects (n = 4). Each data point represents the mean region‐averaged value across subjects with error bars indicating the standard deviation. The black dash line marks the identity line. [file MRM-96-817-s001.docx]

**Mesoscale whole-brain *T*_2_^*^-weighted and associated quantitative MRI in humans at 10.5 T**

Jiaen Liu^1,2,*^, Peter van Gelderen^3^, Jacco A. de Zwart^3^, Jeff H. Duyn^3^, Yujia Huang^1^, Shuxian Qu^4^, Andrea Grant^4^, Edward Auerbach^4^, Matt Waks^4^, Russell Lagore^4^, Lance Delabarre^4^, Alireza Sadeghi-Tarakameh^4^, Yigitcan Eryaman^4^, Gregor Adriany^4^, Kamil Ugurbil^4^ and Xiaoping Wu^4^

1 Advanced Imaging Research Center, UT Southwestern Medical Center, Dallas, TX, USA

2 Radiology, UT Southwestern Medical Center, Dallas, TX, USA

3 Advanced MRI section, NINDS, NIH, Bethesda, MD, USA

4 Center for Magnetic Resonance Research, Radiology, Medical School, University of Minnesota Twin Cities, Minneapolis, MN, USA

* Corresponding author (email address: [jiaen.liu@utsouthwestern.edu](mailto:jiaen.liu@utsouthwestern.edu))

**Supporting information**


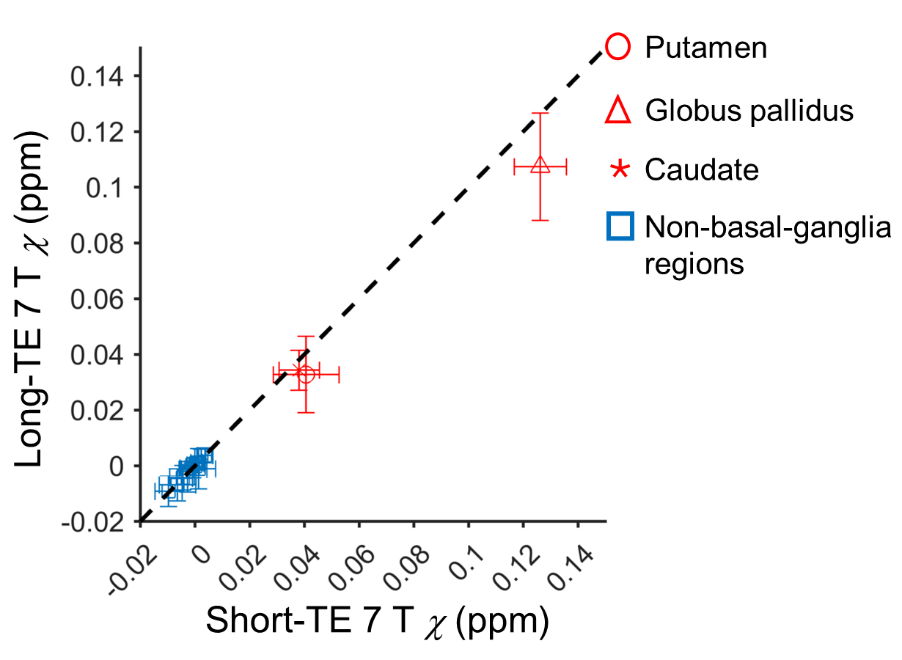


Figure S1. Relation of measured region-averaged 7 T susceptibility (*χ*) reconstructed using short vs. long echo times (TE) in all subjects (n=4). Each data point represents the mean region-averaged value across subjects with error bars indicating the standard deviation. The black dash line marks the identity line.
